# Evaluation of N95 respirators on fit rate, real-time leakage, and usability among Chinese healthcare workers: study protocol of a randomized crossover trial

**DOI:** 10.3389/fpubh.2023.1266607

**Published:** 2023-11-17

**Authors:** Simon Ching Lam, Aderonke Odetayo, Ignatius Tak Sun Yu, Sony Nai Yeung So, Kin Cheung, Paul Hong Lee, Lorna Kwai Ping Suen

**Affiliations:** ^1^School of Nursing, Tung Wah College, Kowloon, Hong Kong SAR, China; ^2^School of Public Health and Primary Care, The Chinese University of Hong Kong, Shatin, Hong Kong SAR, China; ^3^School of Nursing, The Hong Kong Polytechnic University, Kowloon, Hong Kong SAR, China; ^4^Southampton Clinical Trials Unit, University of Southampton, Southampton, United Kingdom

**Keywords:** N95 respirators, fit rate, real-time leakage, usability, Chinese healthcare workers, crossover trial

## Abstract

**Background:**

N95 respirators are used to limit the transmission of respiratory viruses in clinical settings. There are two to three major types of N95 available for all healthcare workers in Hong Kong. However, after the coronavirus outbreak and the consequent shortage of many commonly used respirators, several new N95 respirators were adopted temporarily in clinical settings without evaluation. Prior literature indicates that traditional N95 respirators used in hospitals in Hong Kong are not fit for Chinese people and have fit rates ranging from 50 to 60%. This study aims to investigate and compare the fit rate, real-time leakage, and mask usability of traditional and new N95 respirators among Chinese healthcare workers.

**Methods:**

This study will employ two sequential phases. Phase 1 has a cross-sectional exploratory design used to investigate the fit rate and mask usability of three types of respirators. Phase 2 will examine the effectiveness of respiratory protection by comparing traditional and new N95 respirators by a randomized crossover trial. Eligible participants will be randomly allocated through a controlled crossover experiment to either a traditional or new respirator group (*n* = 100 in each arm) for performing standard clinical procedures. The primary outcome (real-time leakage) will be recorded at 30 s intervals during nasopharyngeal suctioning and cardiopulmonary resuscitation. The secondary outcomes are the fit rate and mask usability. After a 2 min suctioning (15 s twice) and 4 min one-person CPR, the fit rate (assessed by standard N95 fit testing) and mask usability (measured by self-reported mask usability scale) will be recorded as data of post-procedure. After 10 min rest, measurement of real-time leakage (i.e., crossover), fit test, and usability will be repeated.

**Discussion:**

The result of real-time leakage will be a vital indicator of the respiratory protection of Chinese healthcare workers while performing prevalent clinical procedures, such as resuscitation. The fit rate and usability result will serve as an essential reference for consumable purchase policy in clinical settings.

**Trial registration:** ISRCTN registry: ISRCTN40115047. Retrospectively registered on May 9, 2023. https://www.isrctn.com/ISRCTN40115047.

## 1 Introduction

### 1.1 Background and rationale

Worldwide outbreaks of different infectious diseases have caused an increased awareness of occupational protection among healthcare workers (HCWs). Therefore, the use of N95 respirators as an effective nonpharmaceutical measure to limit the spread of diseases in hospitals is recommended by the World Health Organization (WHO) and the Centre for Disease Control and Prevention (CDC) (1, 2). However, regardless of the shape and brand of such respirators, they are all generally tight-fitting half-facepiece type, and their credibility depends on fit to the wearer (3–5). For ill-fitting respirators, the average penetration by ambient aerosol was found to be 33% compared with 4% for well-fitting respirators (6). To achieve creditable occupational protection, most known authorities, such as the National Institute for Occupational Safety and Health (NIOSH), CDC, WHO, and the Hospital Authority (HA) in Hong Kong, made fit testing compulsory for wearers prior to the use of an N95 respirator (1, 3–5, 7).

N95 respirators are used to limit the transmission of respiratory viruses in clinical settings. Hospitals under the HA and private hospitals regularly provide two to three major types of N95 respirators available for all HCWs, particularly for high-risk caring procedures, such as suctioning, working in airborne precaution rooms, and resuscitation (7). Commonly used types of N95 respirators are the cup-shaped 3 M 1860 (regular or small size), cup-shaped 3 M 8210 (one size), and the three-panel designed 3 M 1870+ (one size) (8–10). Although manufacturers claimed that all traditional 3 M respirators are 86–100% fit for people (8–10), their performance when donned for Chinese people is suboptimal in terms of protection (reflected by fit rate and real-time leakage) and comfort (reflected by mask usability) compared with that of Western people (3–5, 7, 11, 12).

After the coronavirus (COVID-19) outbreak and the consequent shortage of many commonly used respirators, several new N95 respirators, such as Alpha Pro Tech N95 and NASK M0011 Professional Edition, were adopted temporarily in clinical settings without comprehensive evaluation (13, 14). NASK M0011 comprises nanofibers and has an ear-loop design with two plastic clips, which differs from the traditional melt-blown nonwoven material and head-loop design. The nanomaterial features ultra-thin, lightweight, and breathable properties, allowing wearers to use it for the whole day comfortably. Tests through respected international laboratories, such as the Nelson Laboratories, confirmed a virus filtration efficiency of >99.9%. The material for and design of NASK was officially adopted in public hospitals on 24 April 2020 (13, 14). However, given such a considerable effect on infection control and prevention, the evaluation of reasonable fit rate, real-time leakage, and usability was not comprehensively checked. Besides, the CDC warned that donning a respirator with ear loops as the head harness is difficult to achieve an adequate fit (2).

Quantitative fit testing is a recognized method to determine whether a tight-fitting respirator fits a wearer. This method adopts an electronic device to measure the ratio of particular air particles inside and outside the breathing zone (when donned with a respirator), and the ratio (i.e., fit factor) reflects the degree of leakage (3–5). Therefore, fit rate (the overall passing rate of fit testing) would be the primary concern to determine the fit of an N95 respirator to a population. Studies on American and Canadian HCWs indicated that several brands and types of respirators developed in the West (e.g., 3 M 1870 and 1860) fit 90–100% (9, 10). However, local retrospective analysis reported the fit rate of two N95 respirators (i.e., 3 M 1860S and 9210) from 84 to 91 nursing staff working in intensive care units (12). The fit rate was as low as 55–69% only. Further local studies examined the fit rate of several commonly used N95 respirators among nursing students and reported a consistently low fit rate of 57.4–65.0% (3, 4). In contrast to the results of studies conducted in the West, the fit rates of respirators were lower when donned by Chinese people. These results may reveal that tight-fitting N95 respirators require a match between mask design and facial anthropometry of a given population (e.g., face length and width) (15). However, the fit rates of all newly adopted N95 respirators for Chinese HCWs are unclear, except for some of the preliminary findings indicating that locally developed respirators (i.e., NASK head-loop model) provide a better-fit rate (78.8%) after nursing procedures (11).

Most of the pre-set quantitative fit testing systems require the wearer with a donned N95 respirator to perform a series of exercises, including a static portion without body movement and a dynamic portion with normal breathing and designated movement (i.e., side-to-side, and up-and-down head movement, talking or reading a standard set of passages, grimacing, and bending over). These exercises stimulate common working activities in the clinical environment (5, 7, 9). Several local studies have challenged the reliability of real-time protection of respirators during nursing procedures based on the sole result of fit testing because performing specific procedures involved a greater range of body movement, such as changing napkin, suctioning, and nasogastric tube insertion (11, 16, 17). Real-time leakage (also named face-seal leakage) was measured by a validated portable aerosol spectrometer (17). It was found that the different particles concentration (size >0.3 μm, >0.4 μm, >1.0 μm, and > 4.0 μm) inside a fit respirator significantly increased before and during suctioning and nasogastric tube insertion (15). Preliminary results suggested that the fit factor of the NASK head-loop model decreased to less than that of the traditional model (11). However, these studies did not consider testing the real-time leakage of a given “fit” respirator under heaviest-duty processes, such as CPR, which is a life-saving clinical procedure, and compulsory use of N95 respirators in hospital settings (18).

Although HCWs assume some level of personal occupational risk when caring for contagious patients, numerous policies and regulations call for respiratory protection in the healthcare environment, noncompliance is unfortunately quite common (19). In general, mask usability is a concept used to reflect conditions, including discomfort, interference with occupational duties, and poor communication experienced by the wearers of N95 respirators (20). Discomfort is often associated with tight-fitting N95 respirator models, including 3 M models used in Hong Kong. Such discomfort encompasses various sensations and experiences, including facial pressure, facial heat, facial pain, labored movement of facial muscles, or skin itchiness (21, 22). Moreover, poor communication has been a concern with existing N95 respirators, as they diminish speech intelligibility in noisy clinical environments (23). During the COVID-19 outbreak, usability can also reflect the maximum duration for the extended use of a given respirator. This parameter is crucial during the pandemic because of the global shortage of personal protective equipment (24). Poor mask usability influences the compliance of N95 respirator use (19) and reduces the applicability for extended use, as some HCWs do not wear N95 respirators as indicated when performing resuscitation during the non-pandemic period.

Literature indicates that traditional N95 respirators used in hospitals in Hong Kong are not fit for Chinese people, having fit rates ranging from 50 to 65% compared with those among Western people (10, 11). Such a result poses a risk of real-time leakage during caring procedures, including suctioning, nasogastric tube insertion, and resuscitation. Our preliminary results found that real-time leakage happens during care procedures requiring moderate body movement, referring to moderate intensity of physical activity. However, the degree of real-time leakage of traditional N95 respirators during procedures requiring high body movement (e.g., CPR) remains unknown. Additionally, changes in fit factors during and after various caring procedures are also unknown. More importantly, new respirators with nanomaterials and ear-loop with clip designs have been introduced in public hospitals. Nevertheless, the fit rate, real-time leakage, and mask usability of such design have not been evaluated, which poses a threat of uncertainty to HCWs. Therefore, this study aims to investigate and compare the fit rate, real-time leakage, and mask usability of traditional and new N95 respirators among Chinese healthcare workers.

## 2 Objectives

### 2.1 Study objectives

This study aims to investigate and compare fit rate, real-time leakage, and mask usability of the traditional and new N95 respirators among Chinese healthcare workers.

#### 2.1.1 Primary objective

To compare real-time leakage by using traditional and N95 respirators during two clinical procedures, which are nasopharyngeal suctioning and CPR.

#### 2.1.2 Secondary objective

To investigate the fit rate and mask usability of traditional and new N95 respirators when donned by Chinese healthcare workers.

## 3 Research hypothesis

We hypothesize that there is no significant group difference in real-time leakage between the use of traditional and new N95 respirators among Chinese healthcare workers during two clinical procedures (i.e., nasopharyngeal suctioning and CPR). We also hypothesize that the fit rate and mask usability have no significant difference between traditional and new N95 respirators among Chinese healthcare workers.

### 3.1 Trial design

The study is a randomized controlled, exploratory crossover trial with two arms (*n* = 100 in each arm) to examine and compare the real-time leakage of traditional and new respirators during two standard clinical procedures (i.e., nasopharyngeal suctioning and CPR).

The study will employ two sequential phases. A cross-sectional exploratory design in phase 1 will investigate the fit rate and usability of three types of respirators (two traditional melt-blown respirators with head-loop design and one new nano-respirator with ear-loop and clips) among Chinese HCWs, which is used to identify potential participants for the next phase. A cross-sectional design is appropriate to estimate the prevalence of unfit respirators through testing in a group of large samples.

**Figure 1:**
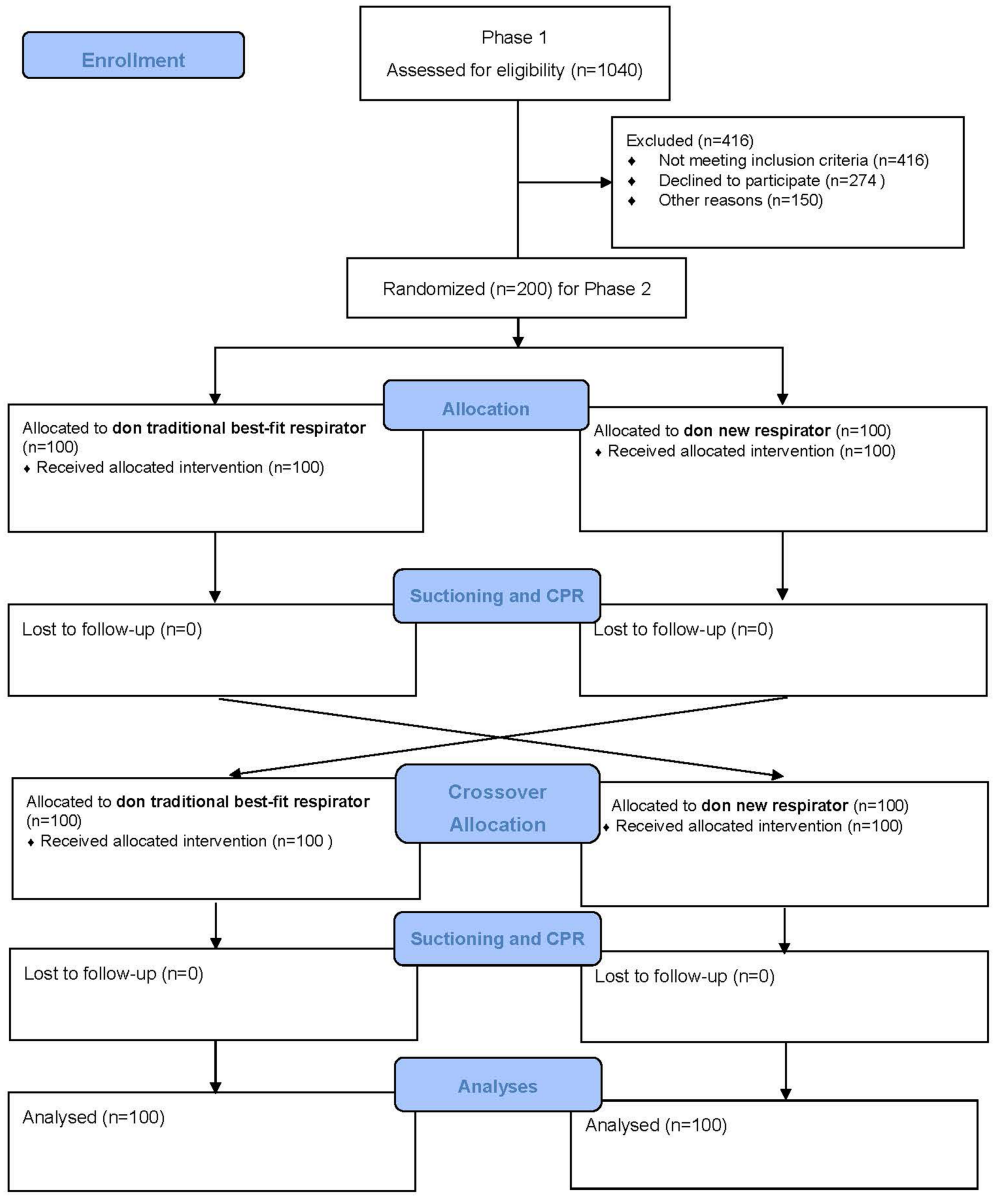
CONSORT flow diagram.

Phase 2 is a randomized controlled crossover trial with two arms: participants donned with traditional best-fit respirators (either 1860S or 1870+), and participants donned with a new respirator (made of nanomaterial and ear-loop with clip design). A best-fit respirator refers to a given respirator obtaining the highest fit factor when donned by a user. A crossover design has advantages over a parallel experiment because the participating subject will act as his or her control, and possible incomparability between “intervention” and “control groups” could be eliminated. A two-arm study can yield reliable evidence about the effectiveness of real-time protection between traditional and new respirators. Randomization is performed as block randomization with a 1:1 allocation. To protect participants’ rights, anonymity, and confidentiality, the study was designed with the ethical principles of the Declaration of Helsinki. The paper complies with the SPIRIT recommendation for study protocol (25). The overall CONSORT flowchart of the study design is shown in Figure 1.

## 4 Method: participants, interventions and outcomes

### 4.1 Study setting

This study is being conducted in Hong Kong. Participants will be approached and recruited from web promotion (40% of samples) and invited collaborators (60% of samples), including HCWs (e.g., 20% doctors and 80% nurses based on the ratio of total doctors to nurses in Hong Kong) working in non-governmental organizations and students studying in healthcare disciplines in universities. These organizations provide HCWs who offer treatment and care to patients directly.

### 4.2 Eligibility criteria

#### 4.2.1 Inclusion criteria

All participants must give written consent for participation following adequate explanation and sufficient understanding before being included in the study.

1. Aged 18 years or above.
2. Healthcare workers who are students or staff (with license) who provide direct care to patients.
3. Certified to perform standard CPR for delivery of basic life support or cardiovascular life support.
4. Have completed an accredited institutional training program and perform CPR in the clinical field.
5. Participants must obtain a pass of fit tests for one traditional N95 respirator (best-fit one) as well as new respirators.

#### 4.2.2 Exclusion criteria

1. Healthcare workers with a history of chronic respiratory diseases or medical conditions (e.g., asthma, congestive heart failure, coronary heart disease, etc.).
2. Pregnant.
3. Having any musculoskeletal diseases that restrict the capacity of CPR performance.

### 4.3 Who will take informed consent?

A research assistant will introduce the trial to the participants by demonstration. Participants will also receive an information sheet. The participants are screened with reference to the inclusion criteria, and eligible participants are invited to join the study using a signed written consent with an explanation. The research assistant will then collect the data of fit testing and mask usability results of all three respirators (two traditional respirators and one new respirator), gender, age, and body weight, which are records required for fit testing. Participants who pass one traditional respirator and one new respirator are invited to the phase 2 study.

### 4.4 Additional consent provisions for collection and use of participant data and biological specimens

Not applicable.

### 4.5 Interventions

#### 4.5.1 Explanation for the choice of comparators

**Figure 2:**
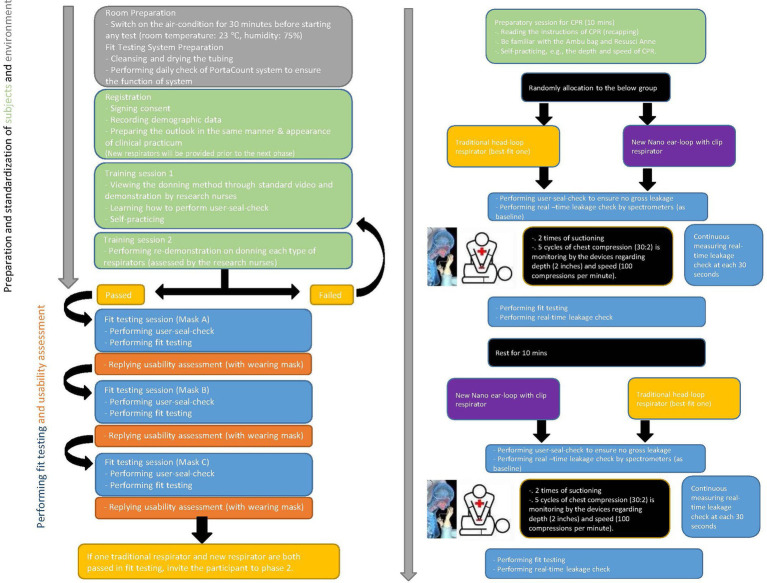
Standardization of N95 respirator-wearing protocol and the flow of crossover trial.

**Figure 3:**
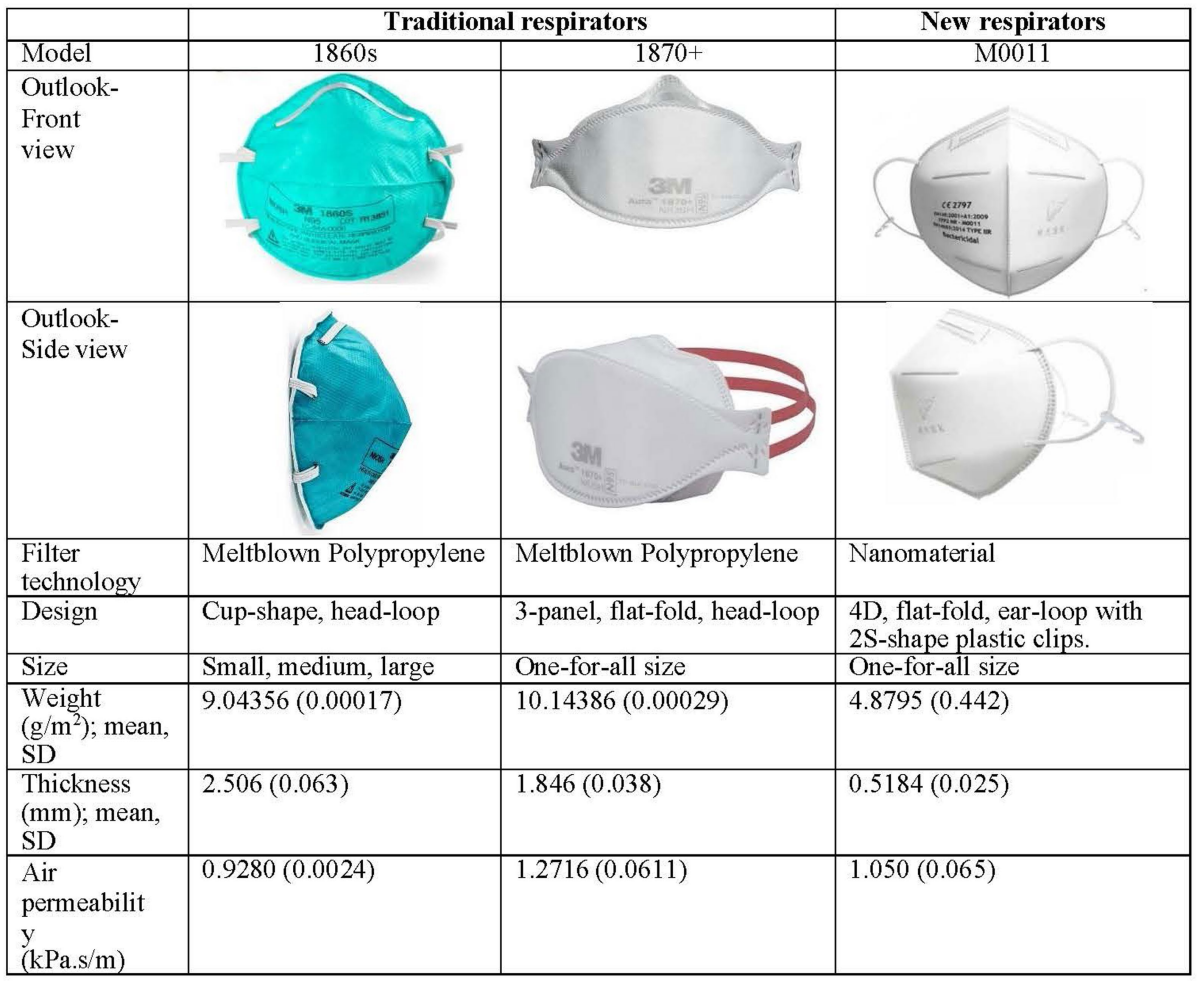
Traditional and new N95 respirators.

To ensure accuracy in comparison of real-time leakage of respirators during CPR, the types of respirators are the infection-control measure serving as an independent variable. Participants receive a traditional best-fit respirator either 3M 1860S or 3M 1870+. All participants are required to don necessary personal protective equipment (assigned N95 respirators, gown, and face shield) for nasopharyngeal suctioning and CPR in the designated simulation environment. Literature indicates that respirator-donning skills, user-seal-check skills, and facial outlook influence the fit-testing results and lead to inaccurate measurement of real-time leakage (3–5). A stringent standardization N95 respirator-wearing protocol is applied (Figure 2), including tying up long hair, standardized psychomotor training on user-seal-check, and standardized psychomotor training on donning skills through video, demonstration, and return demonstration (3–5). Traditional respirators are the commonly used 3 M head-loop respirators in hospitals in Hong Kong, namely the 3 M cup-shaped 1860S and the three-panel-designed 1870+ (7, 8). To the best of our knowledge, these respirators have two of the highest fit rates (3, 5). Participants using these respirators are the control group. New respirators are masks made of nanomaterial and ear-loop with clip design, which have been adopted for clinical use (13). Participants using this new respirator are the intervention group. Comparison of three respirators is presented in Figure 3.

#### 4.5.2 Intervention description

Eligible participants are randomly allocated in equal proportion to either control or intervention (traditional or new respirator group). New respirator as intervention has been illustrated and described in Figure 3. Standard scenarios are introduced to participants in advance to reduce stress from the environment and psychological factors. Sufficient time is provided for participants to familiarize themselves with the devices and settings. Participants are required to don all necessary personal protective equipment (assigned N95 respirators, gown, and face shield) for nasopharyngeal suctioning and CPR in the designated simulation environment. Data of real-time leakage is recorded at 30 s intervals during the nasopharyngeal suctioning and CPR. After 2 min suctioning (15 s for two times) and a 4 min one-person CPR, participants donned with the tested respirator will perform fit testing and respond to mask usability, which is recorded as data of post-procedure. With a 10 min rest, participants will don another respirator and repeat the measurement of real-time leakage (i.e., crossover), fit test, and usability.

#### 4.5.3 Criteria for discontinuing or modifying allocated interventions

Not applicable.

#### 4.5.4 Strategies to improve adherence to interventions and scenarios

A face-to-face adherence session will take place at the designated simulated environments. This will include:

- Instruction to the standardization of N95 respirator-wearing protocol (Figure 2).
- Importance of following instructions for standard scenarios and measurements of the speed and depth of chest compression to ensure minimal discrepancy.
- Instruction about the duration of suctioning (i.e., two times suctioning for a maximum of 15 s each) and one-man CPR (a total of 4 min, which included two series of five cycles) (Figure 2).

#### 4.5.5 Relevant concomitant care permitted or prohibited during the trial

Not applicable.

#### 4.5.6 Provision of post-trial care

Participants enrolled in the study will be monitored by the principal investigator and research nurse continuously to check for problems such as discomfort in performing chest compression, allergic reaction due to the respirators.

### 4.6 Outcomes

#### 4.6.1 Primary outcome measures

Real-time leakage will be measured by portable aerosol spectrometers (Grimm Model 1.109-006 and 007; Grimm Technologies, Ainring, Germany) during two selected clinical procedures. They will provide rapid measurement of particle concentration with optical sizes of 0.25–0.32 μm and different sizes from 31 channels. The system will consist of two spectrometers, the first will measure ambient air particle concentration, and the second will measure air particle concentration inside the respirator. This device will be placed on a designated table throughout the assessment. Participants will perform real-time measurements for real-time leakage at an average of 30 s intervals while performing the standard clinical procedures. A total of eight sets of data are collected within a 4 min procedure while 4 sets of data are collected in a 2-min one. Portable aerosol spectrometers have been used to measure real-time leakage during aerosol-generating procedures and some nursing procedures, namely, open suctioning, nasogastric-tube insertion, and napkin change (15, 16).

#### 4.6.2 Secondary outcome measures

Fit rate and mask usability will be assessed and measured.

**Figure 4:**
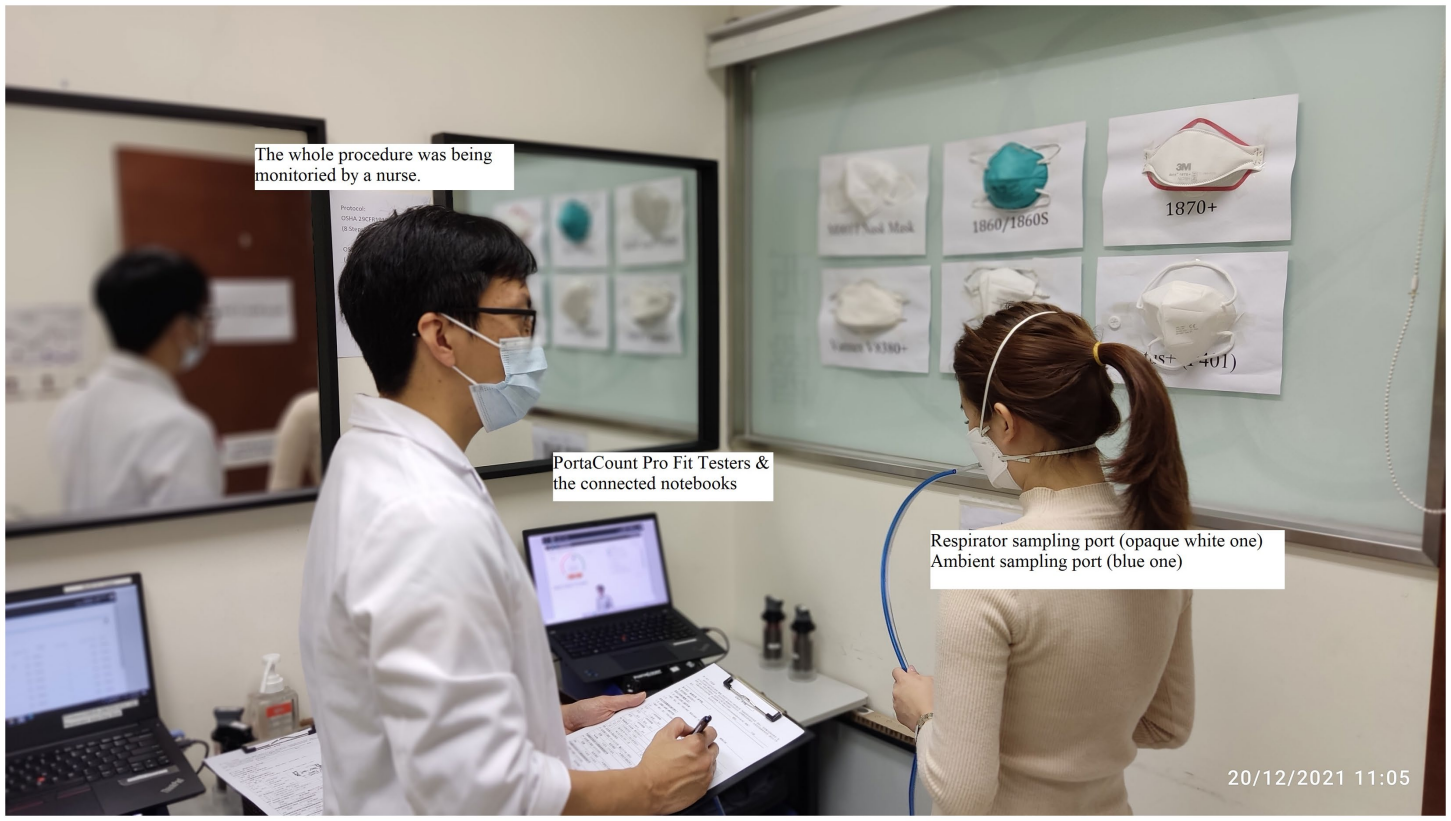
Quantitative fit testing system, tubing connection, and respirator.

Quantitative fit testing will be conducted with a PortaCount respirator fit-tester system (model Pro+8038/8040; TSI limited), a recognized technology for counting air particles. The overall fit factor (range = 0–200) is the ratio of the concentration of a challenge agent (ambient particles) outside the respirator to the concentration of the same agent that leaks into the respirator (26). Fit rate is the proportion of the number of fit factor > 100 among total tested samples with a given respirator. A fit exceeding 100 indicates a passing rate, meaning that a given respirator is well-fitted to the wearer (3, 4, 12, 16, 27) (Figure 4).

Mask usability will be measured by an 11-item Mask Usability Scale (MUS), which involves the wearer’s feedback on tolerated heat, breathability, tightness, interference on speaking, skin itchiness, mask displacement, discomfort on the ear lobe, duration of use, interference on working efficiency, mist over glasses, and overall level of comfort (unpublished data). The MUS is rated on a six-point Likert scale, where a lower score means better usability. We have developed this scale and preliminarily used it in our previous studies (11, 16). We have also validated this scale on 283 nursing students with satisfactory psychometric properties (Cronbach’s alpha = 0.81–0.87; 2-week test–retest reliability: intraclass correlation coefficient = 0.821–0.822).

### 4.7 Sample size

Calculation of sample size in this study was carried out based on Cochran’s formula for sampling size estimation (*N* = Z^2^p(1–p)/d^2^) (28, 29), where Z is 1.96 for a 95% confidence level, p is 40% for the estimated prevalence of unfit respirators (3, 4, 14), and d is 3% for an acceptable margin of error, for determining fit rate and mask usability (29). A quota sampling will be conducted to ensure that the samples include a certain proportion of students and staff (ratio of 1:2). Our previous study indicated that the medium to large effect size on group differences between traditional and new respirators can be obtained using real-time leakage (11). We will assume an effect of 0.40 to achieve a power of 80% at a 5% significance level (30). 1040 HCWs were recruited in phase 1, of which 60% of Chinese HCWs obtained a pass rate of fit testing in either traditional respirators (3, 4, 12, 14). Coupled with those who passed the fit testing for the new respirator (about 79%) as well as the 20% attrition rate, we have sufficient eligible samples to invite for the phase 2 study (1040 × 60% × 78% × 0.8 = 389). A total sample size of 200 will be required for this study.

#### 4.7.1 Participant timeline

**Figure 5:**
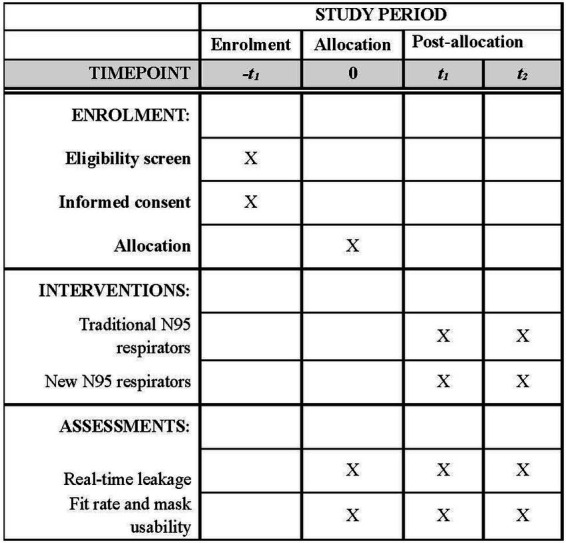
SPIRIT figure of participant timeline.

Refer to Figure 5 for the SPIRIT figure of the participant timeline.

#### 4.7.2 Recruitment

Recruitment takes place at the Integrative Health Center, Tung Wah College, and Squina International Center for Infection Control. These centers are well-equipped with fit testing devices and simulated clinical environments for performing CPR. The devices and facilities have been accredited and used for practical examination by the Hong Kong Nursing Council.

### 4.8 Assignment of interventions: allocation

#### 4.8.1 Sequence generation

The randomization process will be conducted using computer-generated randomization by a research nurse to determine random allocation, that is the sequence of donning the respirators in this crossover design. All participants will receive an anonymous identifying number to ensure anonymity.

Participants will be randomized with equal probability (1:1 ratio). The block sizes will not be disclosed to ensure concealment.

#### 4.8.2 Concealment

Participants will be randomized using sealed envelopes. Only the research nurse who generated the allocation sequence knows the order of randomization.

#### 4.8.3 Implementation

Participants are randomly allocated to either the group using a traditional respirator or to the group using the new respirator according to an allocation sequence prepared by the research nurse before the start of the study.

### 4.9 Assignment of interventions: blinding

#### 4.9.1 Who will be blinded

The research team member assigned to perform data input is blinded to the allocation sequence.

#### 4.9.2 Procedure for unblinding if needed

Not applicable.

## 5 Data collection and management

### 5.1 Plans for assessment and collection of outcomes

The plan for the collection of outcomes is illustrated in Figure 2 and the measurements are described in the abovementioned section of “Outcomes.”

### 5.2 Plans to promote participant retention and complete follow-up

Once a participant is enrolled or randomized, the principal investigator will ensure reasonable effort to follow the participant through the study period.

Participants may withdraw from the study for any reason at any time, without consequences. Participants may also withdraw if the study sponsor or organization sponsoring the trial terminates the study before the proposed end date. Participants who complete the trial are provided with HK$300 (~US$38) incentive as time compensation.

### 5.3 Data management

All data recorded are stored and backed up on an encrypted hard drive. Data entry and management will be completed by one of the research team members and double-checked by a minimum of one other team member. Personal identifiers will be removed upon completion of the study and stored for long-term retention of the research data. Data will be retained for at least 7 years.

### 5.4 Confidentiality

Participant and study-related information will be securely stored at the study site with limited access. All data collection and administrative forms will be coded to maintain participant confidentiality. Personal identifiers, such as informed consent forms will be removed from the local database and stored separately from participant information.

### 5.5 Plans for collection, laboratory evaluation and storage of biological specimens for genetic or molecular analysis in this trial/future use

Not applicable.

## 6 Statistical methods

### 6.1 Statistical methods for primary and secondary outcomes

Statistical Package for the Social Sciences (SPSS) version 27 will be used to perform descriptive and inferential statistical analysis. Descriptive statistics, including mean, standard deviation, percentage, and frequency, will be used to report the demographics, fit rate, and mask usability. The intention-to-treat (ITT) principle will be applied. We expect no carry-over and no period effect for this study based on previous experience. Chi-square test for categorical variables and independent sample t-test for continuous variables will be used to examine comparability among groups by randomization. Independent sample *t*-tests and paired *t*-tests will be used to compare real-time leakage among different groups of tested respirators during procedure, before and after procedures, respectively. Generalized estimating equations (GEE) will be used to evaluate the total real-time leakage difference in the interaction of time and cycles of chest compression. All the tests will be two-sided, and a *p*-value will be set at 0.05 for statistical significance.

### 6.2 Interim analyses

Interim analysis is performed by a statistician, blinded to the allocation sequence. The interim analysis is reported to the monitoring committee. The monitoring committee will discuss the results of the interim analysis with the steering committee in a joint meeting. The continuation of the trial is decided by the steering committee and will be reported to the Ethics Committee.

### 6.3 Methods of additional analyses (e.g., subgroup analyses)

Not applicable.

### 6.4 Methods in analysis to handle protocol non-adherence and any statistical methods to handle missing data

Not applicable.

### 6.5 Plans to give access to the full protocol, participant level-data and statistical code

Not applicable.

## 7 Oversight and monitoring

### 7.1 Composition of the coordinating centre and trial steering committee

#### 7.1.1 Principal investigator

The principal investigator will prepare the protocol and revisions, collaborate with participating institutions to secure efficient participant recruitment and organize steering committee meetings once a month. The principal investigator is responsible for training research assistant and research nurse, and monitoring data collection procedures. Additionally, the principal investigator is responsible for the publication of study reports.

#### 7.1.2 Steering committee

The steering committee consists of the principal investigator, co-investigators, frontline nursing officer, occupational health and aerosol protection expert, occupational health physician, and statistician. All lead investigators will be steering committee members and will have an agreement on the final protocol. The committee is responsible for participant recruitment, monitoring the trial’s overall conduct, and protecting its credibility.

#### 7.1.3 Trial management committee

This committee led by the principal investigator meets every month at the study site. The committee reviews the progress of the study, and if necessary, agrees to changes to the protocol.

### 7.2 Composition of the data monitoring committee, its role and reporting structure

A data monitoring committee (DMC) has been established. The study organizers are not affiliated with the DMC. During recruitment to study, interim analyses will be given to DMC in strict confidentiality. The DMC will provide guidance to the trial steering committee based on the interim evaluations.

### 7.3 Adverse event reporting and harms

Not applicable.

### 7.4 Frequency and plans for auditing trial conduct

The study is monitored by the Sponsor per the approved protocol alongside the relevant regulatory body. Monitoring occurs bi-yearly, and a review of data collection is carried out to ensure accurate and complete data reports.

### 7.5 Plans for communicating important protocol amendments to relevant parties (e.g., trial participants, ethical committees)

Any modification to the protocol or administrative changes that may impact the conduct of the study will require formal amendment of the protocol. This amendment will be agreed upon by the Research Fund Secretariat of Health Bureau and approved by the Ethics Committee before implementation.

### 7.6 Dissemination plans

Local dissemination of research findings will be to the Infection Control Branch of the HA Hospital Authority and the Centre of Health Protection of the Hong Kong SAR Government for their information and reference. The study results will be disseminated at local and international conferences and in refereed journals related to infection control and occupational health, aiming to draw worldwide attention to this real-time leakage issue and popularize the importance of facial anthropometrics in N95 respirator design.

## 8 Discussion

N95 respirators are used to limit the transmission of respiratory viruses in clinical settings. This randomized crossover study will serve as an essential reference for occupational safety as well as consumable purchase policy in clinical settings. The fit rate and usability result will be informative regarding which type of N95 respirators best fits most Chinese HCWs in terms of passing rate of fit testing and usability. The results of real-time leakage will be vital indicators of the respiratory protection provided to HCWs during their performance of prevalent aerosol-generating procedures, like suctioning and resuscitation. The selection of these clinical procedures is because they are common aerosol-generating procedures in clinical settings and can represent mild to vigorous intensities of physical activity, which enriches the comprehensive understanding of leakage conditions in relation to the intensity of physical activity. If the new nano-respirator is better than the traditional one for Chinese people, this evidence will reduce the use of many different kinds of respirators and the time used for fit testing because of failed fit testing. It will also increase real-time protection during clinical procedures and enhance compliance with donning N95 respirators because of better usability. All of the above clinical implications as nonpharmaceutical interventions to limit the spread of infectious respiratory pathogens in hospitals.

It is possible that the new nano N95 respirator (i.e., NASK ear-loop design with clips) is not superior to the conventional ones. The results will inform us about what procedures require caution because of real-time leakage, which is beneficial to the occupational health of frontline HCWs. However, we know that the NASK has another N95 head-loop model, which has not yet been verified in any clinical trial. Moreover, several newly developed N95 respirators have recently been developed, such as V8380+ and V9580+, with filed patents licensed from the Hong Kong Polytechnic University. The preliminary fit rate and usability are attractive, and these respirators still need to be fully evaluated. However, these respirators haven’t been fully adopted in current public clinical settings. The results could provide a good reference to inform whether we should test other potential respirators for Chinese healthcare workers.

If the new N95 respirators have a better fit rate, greater mask usability, and less real-time leakage, increasing the production of respirators for Chinese HCWs to replace all traditional models is worthy of consideration. Our findings will also provide vital insight to encourage local manufacturers to develop more N95 respirators.

Some limitations deserve discussion. Firstly, this trial does not include all available N95 respirators in the market. The focus of this trial is not to promote some respirators better than others. Instead, the risk of real-time leakage of the use of N95 respirators should be verified for the specific design (e.g., ear-loop with clips) or shape (e.g., vertical flat-fold). Secondly, this trial does not include all aerosol-generating procedures in clinical settings but two common procedures are selected to simulate a range of intensity of physical activity. Hence, the results were impossible to generalize to all aerosol-generating procedures. Lastly, the current experiment is conducted through standard scenarios in a controlled simulation environment with mannequins. It is anticipated that the results of real-time leakage may be underestimated or regarded as minimal because the on-site clinical situations in resuscitation consist of many uncontrolled factors and different involved personnel.

### 8.1 Trial status

The trial is completed. However, it is confirmed that the study protocol has not been changed or revised anywhere. The results related to this trial have not been disseminated. Recruitment began in November 2021 and was completed on 31 May 2023. Protocol version 1 was approved by the Ethical Committee on 3 June 2021 and 19 January 2022, respectively.

## Ethics statement

The studies involving humans were approved by Hong Kong Polytechnic University Institutional Review Board Tung Wah College Research Ethics Committee. The studies were conducted in accordance with the local legislation and institutional requirements. The participants will provide their written informed consent to participate in this study. Written informed consent was obtained from the individuals for the publication of any identifiable images or data included in this article.

## Author contributions

SL: Conceptualization, Data curation, Funding acquisition, Investigation, Methodology, Project administration, Writing – original draft, Writing – review & editing. AO: Visualization, Writing – review & editing. IY: Funding acquisition, Methodology, Writing – original draft. SS: Funding acquisition, Writing – original draft. KC: Funding acquisition, Investigation, Methodology, Writing – original draft. PL: Formal analysis, Funding acquisition, Methodology, Writing – original draft. LS: Conceptualization, Funding acquisition, Methodology, Project administration, Supervision, Writing – original draft, Writing – review & editing.
