# Corrigendum: Evaluation of N95 respirators on fit rate, real-time leakage, and usability among Chinese healthcare workers: study protocol of a randomized crossover trial

**DOI:** 10.3389/fpubh.2024.1367687

**Published:** 2024-01-30

**Authors:** Simon Ching Lam, Aderonke Odetayo, Ignatius Tak Sun Yu, Sony Nai Yeung So, Kin Cheung, Paul Hong Lee, Lorna Kwai Ping Suen

**Affiliations:** ^1^School of Nursing, Tung Wah College, Kowloon, Hong Kong SAR, China; ^2^School of Public Health and Primary Care, The Chinese University of Hong Kong, Shatin, Hong Kong SAR, China; ^3^School of Nursing, The Hong Kong Polytechnic University, Kowloon, Hong Kong SAR, China; ^4^Southampton Clinical Trials Unit, University of Southampton, Southampton, United Kingdom

**Keywords:** N95 respirators, fit rate, real-time leakage, usability, Chinese healthcare workers, crossover trial

In the published article, there was an error in the Funding statement. The Funding statement for the article erroneously was displayed as “Health and Medical Research Fund (Research Fund Secretariat, Research Office, Health Bureau, Hong Kong ref no: HMRF210401)”. The correct Funding statement is “The Health and Medical Research Fund, the Health Bureau, The Government of the Hong Kong Special Administrative Region (Ref No. 20190322)”.

The authors apologize for this error and state that this does not change the scientific conclusions of the article in any way. The original article has been updated.

